# Tet1 Regulates Astrocyte Development and Cognition of Mice Through Modulating GluA1

**DOI:** 10.3389/fcell.2021.644375

**Published:** 2021-10-28

**Authors:** Weize Xu, Xicheng Zhang, Feng Liang, Yuhang Cao, Ziyi Li, Wenzheng Qu, Jinyu Zhang, Yanhua Bi, Chongran Sun, Jianmin Zhang, Binggui Sun, Qiang Shu, Xuekun Li

**Affiliations:** ^1^The Children’s Hospital, School of Medicine, Zhejiang University, Hangzhou, China; ^2^National Clinical Research Center for Child Health, Hangzhou, China; ^3^The Second Affiliated Hospital, School of Medicine, Zhejiang University, Hangzhou, China; ^4^The Institute of Translational Medicine, School of Medicine, Zhejiang University, Hangzhou, China; ^5^Department of Biostatistics, The University of Texas MD Anderson Cancer Center, Houston, TX, United States; ^6^Department of Neurobiology and Department of Neurology of the First Affiliated Hospital, Zhejiang University School of Medicine, Hangzhou, China

**Keywords:** astrocyte, Tet1, neuronal development, cognition, GluA1

## Abstract

Tet (Ten eleven translocation) family proteins-mediated 5-hydroxymethylcytosine (5hmC) is highly enriched in the neuronal system, and is involved in diverse biological processes and diseases. However, the function of 5hmC in astrocyte remains completely unknown. In the present study, we show that *Tet1* deficiency alters astrocyte morphology and impairs neuronal function. Specific deletion of *Tet1* in astrocyte impairs learning and memory ability of mice. Using 5hmC high-throughput DNA sequencing and RNA sequencing, we present the distribution of 5hmC among genomic features in astrocyte and show that *Tet1* deficiency induces differentially hydroxymethylated regions (DhMRs) and alters gene expression. Mechanistically, we found that *Tet1* deficiency leads to the abnormal Ca^2+^ signaling by regulating the expression of GluA1, which can be rescued by ectopic GluA1. Collectively, our findings suggest that Tet1 plays important function in astrocyte physiology by regulating Ca^2+^ signaling.

## Introduction

As the most abundant glial cells in the central nervous system (CNS), astrocytes are involved in regulating the physiology and pathology of the CNS, such as maintaining CNS homeostasis (Allen and Lyons, 2018). Neurogenesis refers to the proliferation of neural stem cells, lineage commitment, morphological development, and synaptic integration of newborn neurons (Song et al., 2002; Sultan et al., 2015; Cope and Gould, 2019). Astrocytes can also regulate synaptic information processing by releasing signaling molecules, such as transmitters, ATP, as well as trophic factors (Harada et al., 2015; Santello et al., 2019). Consequently, the dysfunction of astrocyte can result in behavioral deficits and involves multiple neurodevelopmental and neurodegenerative diseases (Molofsky et al., 2012; Chung et al., 2015; Phatnani and Maniatis, 2015; Sofroniew, 2015; Zuchero and Barres, 2015; Allen and Lyons, 2018; Santello et al., 2019; Valori et al., 2019). Both Rett syndrome and fragile X syndrome are neurodevelopmental disorders caused by mutation of *MeCP2* and *FMR1*, respectively. *MeCP2*- or *FMR1*-deficient astrocytes induce abnormal neuronal development, while the restoration of mutant genes in astrocytes can ameliorate behavioral deficits of mice (Ballas et al., 2009; Jacobs and Doering, 2010; Lioy et al., 2011). Furthermore, under some pathological conditions, astrocytes can be reactivated (reactive astrogliosis) and is involved in neurodegenerative diseases (Valori et al., 2019).

Tet (Ten-eleven translocation) family proteins including Tet1, Tet2, and Tet3 mediate the 5-hydroxymethylcytosine (5hmC) modification, which serves as a stable epigenetic marker. Emerging evidences have indicated that 5hmC mediated epigenetic modification regulates neuronal activity, neurogenesis, and cognition and is involved in multiple neurological disorders including autism, Rett syndrome, FXTAS, Alzheimer’s disease, and Huntington’s disease (Mellen et al., 2012; Tan and Shi, 2012; Kaas et al., 2013; Rudenko et al., 2013; Wang et al., 2013; Zhang et al., 2013; Yao et al., 2014; Papale et al., 2015; Shu et al., 2016; Li X. et al., 2017). Tet1, one of the three Tet protein members, is abundant in mouse brain. Constitutive deficiency of *Tet1* leads to deficits of learning and memory by regulating neuronal gene expression (Kaas et al., 2013; Rudenko et al., 2013; Zhang et al., 2013). Given these results are collected with constitutive *Tet1* KO mice, astrocyte Tet1 may contribute to these phenotypes.

Emerging evidence has shown that DNA modification regulates the function of astrocytes (Neal and Richardson, 2018). The expression of astrocytes marker GFAP can be regulated by 5-methylcytosine (5 mC) (Takizawa et al., 2001). Modulating 5-hmC alters the proliferation and lineage commitment of neural stem cells (Zhang et al., 2013; Li X. et al., 2017). In brain cancer glioblastoma, N^6^-methyladenine DNA (6 mA) modifications increase remarkably and are involved in cell proliferation of glioblastoma stem cells (Xie et al., 2018). However, the roles of Tet in regulating the function of astrocytes remains completely unknown.

In the present study we found that *Tet1* depletion significantly reduced the global level of 5hmC in astrocytes and that specific depletion of *Tet1* in astrocytes impaired the learning and memory capabilities of mice. *Tet1* deficiency altered the morphology of astrocytes and led to abnormal neuronal development and aberrant Ca^2+^ signaling in astrocytes. *Tet1* deletion induced differentially hydroxymethylated regions (DhMRs) and altered gene expression. Furthermore, *Tet1* deficiency significantly decreased the expression of GluA1 in astrocytes, and ectopic expression of GluA1 partially rescued the deficits of Ca^2+^ signaling in *Tet1* deficient astrocytes. Our results revealed the essential role of astrocyte Tet1 in regulating neuronal development and cognitive function in mice.

## Results

### *Tet1* Deletion Decreases 5hmC Level in Astrocytes and Impairs the Learning and Memory of Mice

**FIGURE 1:**
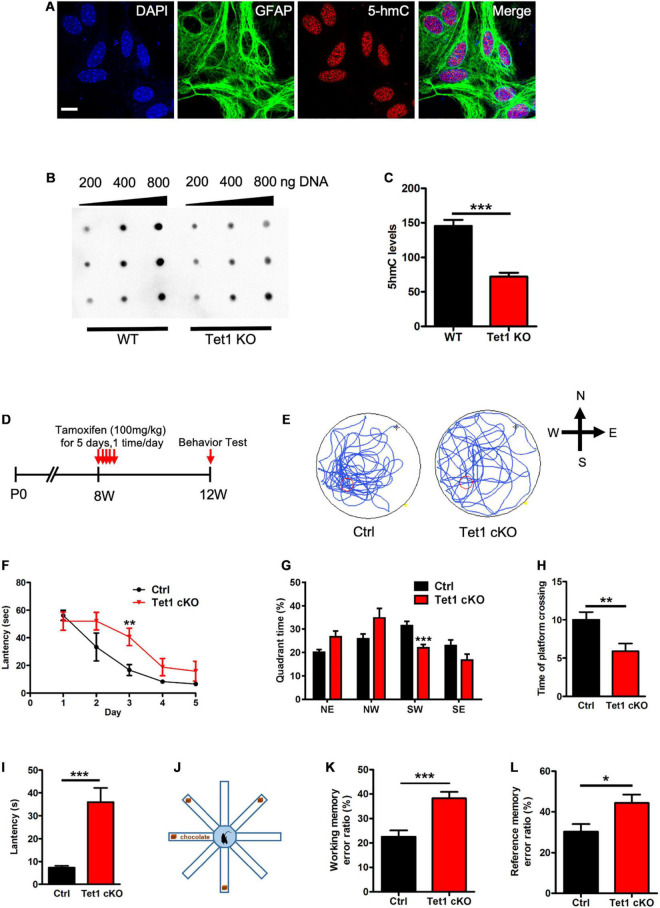
*Tet1* KO reduced the 5hmC level of astrocyte and impaired the learning and memory of mice. **(A)** Representative immunostaining (IF) images of primary astrocyte with nuclei dye DAPI, astrocyte marker Gfap, and 5hmC. Scale bar, 20 μm. **(B,C)** Representative dot blot images with 5hmC-specific antibody **(B)** and quantification showed that *Tet1* KO significantly decreased the level of 5hmC. *n* = 3 for each group. Data were presented as mean ± SEM, unpaired *t*-test; *P < 0.05; **P < 0.01; ***P < 0.001. **(D)** Diagram illustrates the scheme of Tamoxifen (TAM) injection to generate *Tet1* cKO mice. Glast-CreER^*T*2^:Tet1^loxp/lxop^ male mice received 100 mg/kg TAM, or equal volume of solvent as WT, for five consecutive days at the age of 8-week. 4 weeks after the final TAM injection, mice were used for further experiments. **(E)** The latency of WT and cKO mice during five training days. **(F)** Representative images of the swimming path of WT and cKO mice in Morris water maze (MWM) test. **(G–I)** The percentage of time in the quadrants **(G)**, the number of platform crossings **(H)**, and latency **(I)** of WT and *Tet1* cKO mice in the probe test. Data were presented as mean ± SEM, WT = 9, *Tet1* cKO = 11, unpaired *t*-test; *P < 0.05; **P < 0.01; ***P < 0.001. **(J)** The schematic diagram of 8-arm maze test. **(K,L)** Eight-arm maze test showed that cKO mice had higher error ratios for the working memory **(K)** and reference memory **(L)**. Data were presented as mean ± SEM, WT = 9, Tet1 cKO = 11, unpaired *t*-test; *P < 0.05; **P < 0.01; ***P < 0.001.

To examine the function of Tet1 in astrocytes, we first isolated astrocytes from newborn pups (postnatal day 1, P1) of wild-type (WT) and *Tet1* constitutive knockout (KO) mice. Immunofluorescence staining results showed that the cultured astrocytes were positive for astrocyte markers Aldh1l1 and Glast, but not positive for neuronal cell markers Map2 and Tuj1 (Supplementary Figures 1A,B), suggesting a high homogeneity of cultured astrocytes. *Tet1* mRNA was almost non-detectable in *Tet1* KO astrocyte (Supplementary Figure 1C). We next performed 5hmC immunofluorescence staining and found that 5hmC was localized in the nuclei of Gfap positive (Gfap +) astrocytes (Figure 1A). DNA dot blot with 5hmC specific antibody and quantification results showed a significant decrease of global 5hmC level in KO astrocyte compared to WT astrocyte (Figures 1B,C). A representative image of methylene blue staining indicated the equal amount loading of DNA in WT and KO samples (Supplementary Figure 1D).

To specifically delete Tet1 in astrocytes, adult (postnatal 8-week-old) *Glast*-CreER^T2^:*Tet1*^loxp/f^ mice were injected with tamoxifen (i.p.) and sunflower oil, respectively (Figure 1D). A Morris water maze test showed that *Tet1* cKO mice spent shorter time in the target quadrant, crossed the platform in less numbers, and had increased escape latency, but showed no difference in swimming speed and distance (Figures 1E–I and Supplementary Figures 1E,F). We further performed an eight-arm maze test (Figure 1J) and found that cKO mice also displayed higher error ratios of working memory and reference memory (Figures 1K,L and Supplementary Figures 1G,H). Taken together, these data indicate specific deletion of *Tet1* in astrocyte impairs the learning and memory of mice.

### *Tet1* Deficiency Inhibit Morphological Development of Astrocyte

**FIGURE 2:**
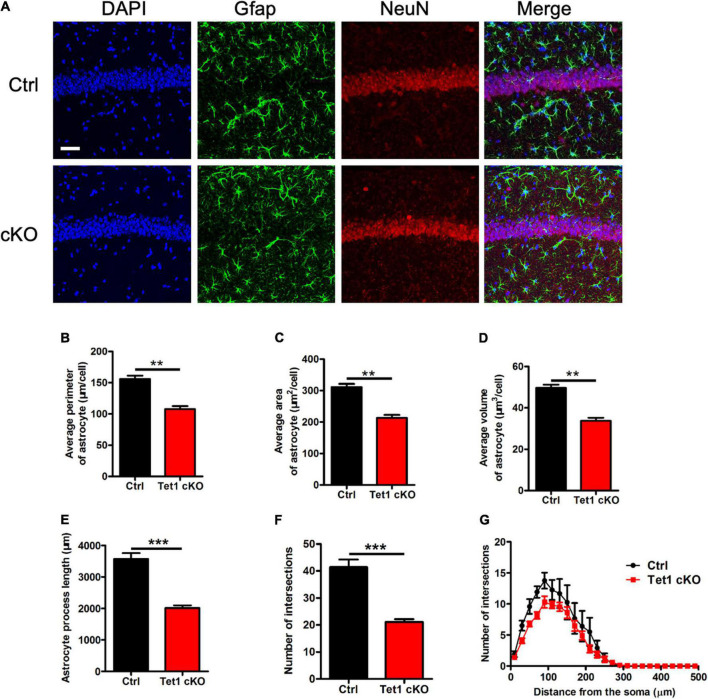
*Tet1* deficiency reduces the complexity of astrocyte morphology. **(A)** Representative immunostaining images of astrocyte marker Gfap and mature neuron marker NeuN with brain sections containing the hippocampus CA1 region of WT and Tet1 cKO mice. Scale bar, 100 μm. **(B–G)** Quantification with Imaris software showed that the perimeter **(B)**, the area **(C)**, the volume **(D)**, the process length **(E)**, the total number of intersections **(F)**, the intersection number at distance from soma **(G)** of astrocyte decreased in Tet1 cKO mice compared to those of Ctrl mice. 5–6 brain sections with the target region were picked up from 3 WT and cKO mice, respectively. Data were presented as mean ± SEM, unpaired *t*-test; *P < 0.05; **P < 0.01; ***P < 0.001.

Next, we performed immunofluorescence staining of astrocyte specific marker Gfap and found that the intensity of Gfap signal was decreased in the hippocampus of cKO mice compared with that of control mice (Figure 2A). Quantification results showed that *Tet1* deficient astrocyte displayed smaller size, shorter length of neurites, and fewer intersections compared to control groups (Figures 2B–G). Immunostaining of another astrocyte marker s100β also showed the decreased signal intensity in the hippocampus of cKO mice compared with that of control mice (Supplementary Figures 2A,B). Collectively, these results suggest that *Tet1* deficiency alters the morphology of astrocytes.

**FIGURE 3:**
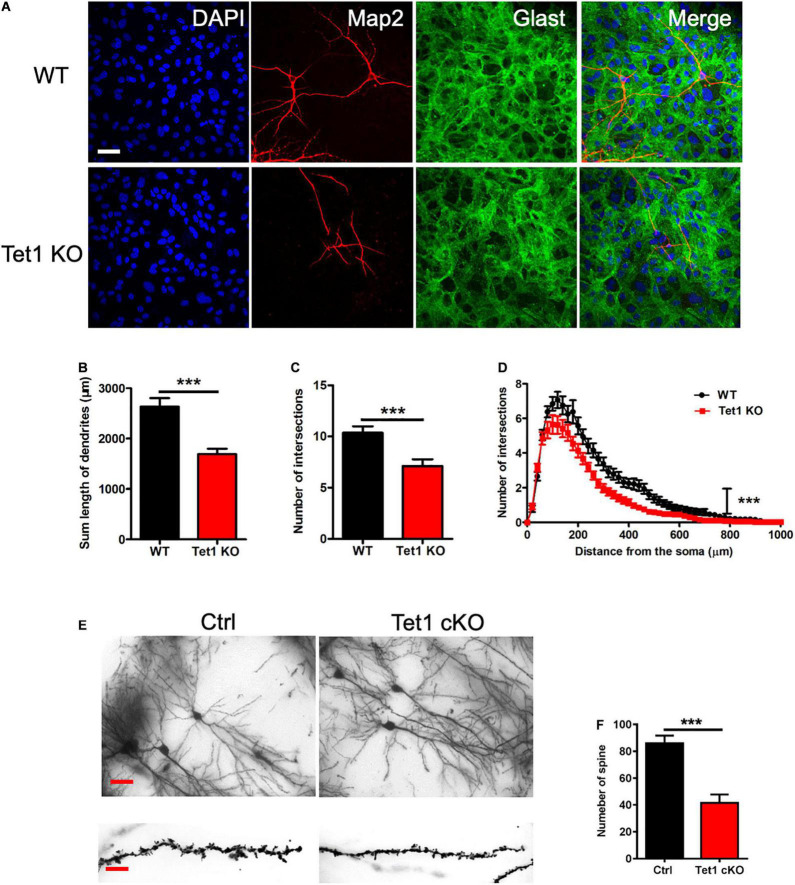
*Tet1* deficiency in astrocyte impairs the morphology of neurons *in vivo* and *in vitro*. **(A)** Representative immunostaining images of astrocyte marker Glast and neuronal cell marker Tuj1. WT hippocampal neurons were co-cultured with WT and KO astrocyte, respectively. Scale bar, 100 μm. **(B–D)** Quantification results showed that neurons co-cultured with KO astrocyte had the decreased dendritic length **(B)** and number of intersections **(C)**. Sholl analysis showed that neurons co-cultured with KO astrocyte also had the reduced intersection number at distance from soma compared to neurons co-cultured with WT astrocyte. 42 neurons co-cultured with WT astrocytes and 36 neurons co-cultured with *Tet1* KO astrocytes were analyzed, respectively. Data were presented as mean ± SEM, unpaired *t*-test; ***P < 0.001. **(E)** Representative images of Golgi staining with the brain sections of Ctrl and *Tet1* cKO mice. shows the morphology of neuron of WT and Tet1cKO mice in CA1 region. Scale bars, 200 μm (in the upper panels), and 10 μm (in the lower panels). **(F)** Quantification results showed the decreased spine number of neurons in *Tet1* cKO mice compared to Ctrl mice. 10 neurons from Ctrl mice and 9 neurons from cKO mice were analyzed, respectively. Data were presented as mean ± SEM, unpaired *t*-test; ***P < 0.001.

To examine the effects of *Tet1* deficiency in astrocyte on neuronal cells, we adopted a neuron-astrocyte co-culture system (Supplementary Figure 2C). We found that hippocampal neurons co-culturing with *Tet1* KO astrocytes exhibited immature morphology compared to control groups (Figure 3A). Quantification results indicated that hippocampal neurons co-culturing with KO astrocytes displayed shorter dendrites, fewer intersections, and reduced complexity (Figures 3B–D). Of note, the morphology of WT astrocytes became more mature and much larger during the co-culture with neurons, whereas KO astrocytes did not show observable changes (Supplementary Figures 2D–E). Furthermore, Golgi staining and quantification results showed that the neurons in the hippocampus of adult cKO mice showed shorter dendrites and fewer spines compared to WT mice (Figures 3E,F). Taken together, these results suggest that the deficiency of *Tet1* in astrocyte not only alters the morphology of astrocytes but also inhibits the neuronal development *in vitro* and *in vivo*.

### *Tet1* Loss Alters Gene Expression and Leads to Dynamic 5hmC Modification in Astrocytes

**FIGURE 4:**
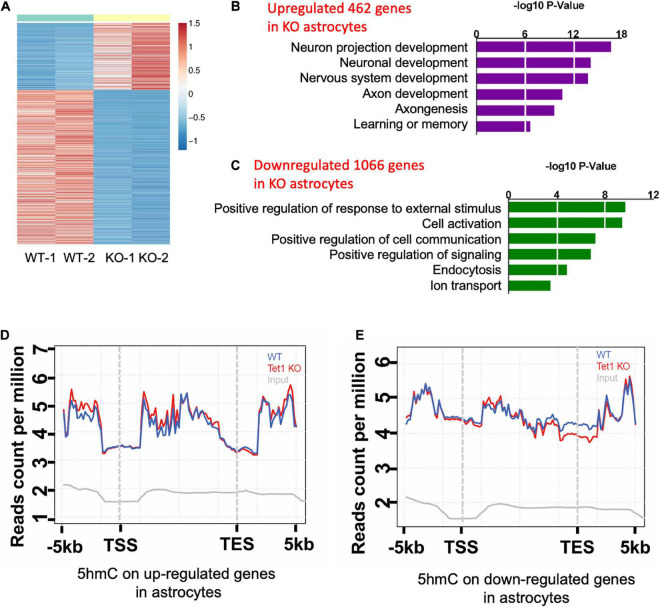
*Tet1* deficiency leads to the altered gene expression and differential hydroxymethylation. **(A)** Heat map drawn from the altered transcriptome of WT and *Tet1* KO astrocytes. Three biological repeats of WT and Tet1 KO cells were adopted for RNA-seq, respectively. The significance of expression was determined by | FC| > 1 and *P*-value < 0.05. **(B,C)** Gene ontology (GO) analysis showed that up-regulated genes enriched for the terms relating with negative regulation of neuronal development and neurogenesis, etc. **(B)**, and down-regulated genes enriched from gliogenesis and cognition, etc. **(D,E)** Averaged 5hmC level over the up-regulated genes **(D)** and down-regulated genes **(E)** of wildtype (WT), Tet1 KO and input astrocyte samples. Tet1 KO astrocyte showed higher 5hmC enrichment on the promoters and gene bodies of up-regulated genes, and lower enrichment on promoters and transcription start sites (TSS) of down-regulated genes.

To reveal the mechanism of astrocyte Tet1 in regulating neuronal development and cognitive function, we next performed RNA-sequencing (RNA-seq) to examine mRNA expression in cultured WT and *Tet1* KO astrocytes. FPKM of astrocyte markers *Gfap*, *Aldh1l1* showed a high level, but the expression markers for neural precursor/stem cell marker *Nestin*, neuronal progenitor cell marker *DCX*, neuronal markers *Map2* and *Tuj1* were almost non-detectable in both WT and KO cells, suggesting a high homogeneity of cultured astrocytes (Supplementary Figure 3A). The results of RNA-seq showed that a total of 4,116 genes showed altered expression in KO astrocytes compared to WT cells: 1,943 up-regulated and 2,173 down-regulated (*P* < 0.05, fold change > 1) (Figure 4A and Supplementary Table 1). Gene Ontology (GO) analysis of the altered genes showed enrichment of genes related to neuronal development, axon development, response to external stimulus, cell activation, and ion transport, etc. (Figures 4B,C).

Next, we performed genome-wide 5hmC profiling of WT and *Tet1* KO astrocytes utilizing an established 5hmC chemical labeling and affinity purification method (Song et al., 2011; Szulwach et al., 2011). We analyzed 5hmC sequencing data with an established pipeline and found that 5hmC was highly enriched in distinct genomic regions, such as intron, exon, promoter, and intergenic regions in WT astrocytes. We also found that *Tet1* KO did not significantly alter the distribution landscape of 5hmC in the genome (Supplementary Figures 3B,C). Further, the differential hydroxymethylation regions (DhMRs) induced by *Tet1* KO were enriched in intron, exon, promoter, and intergenic regions (Supplementary Figures 3D, 4E), which were associated with 9,547 genes (Supplementary Table 2). We further performed a correlation analysis of DhMRs and genes with altered expression indicated by RNA-seq. Our results revealed increased enrichment of 5hmC distribution on up-regulated genes, especially at promoter and gene body regions (Figure 4D), and decreased 5hmC distribution on down-regulated genes, especially at promoter, and TSS regions (Figure 4E). These results suggest a positive correlation of 5hmC and gene expression in astrocytes.

### *Tet1* Deficiency Leads to Down-Regulation of *GluA1* and Induces Abnormal Ca^2+^ Signaling

**FIGURE 5:**
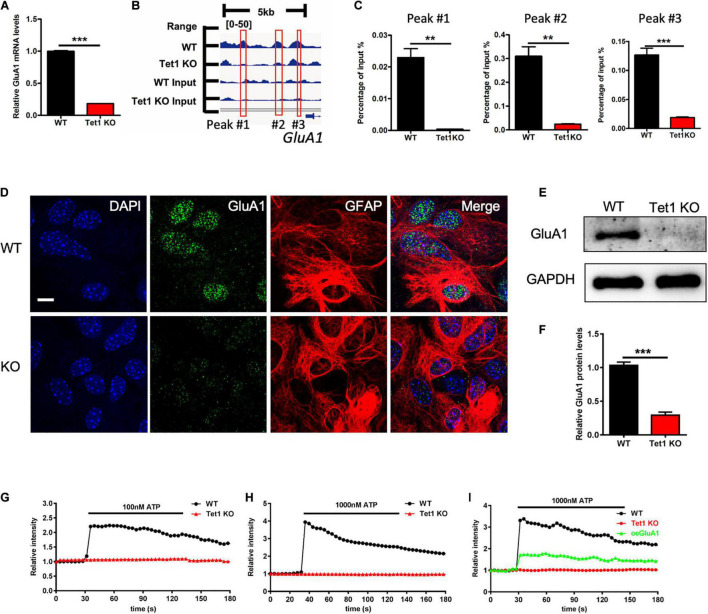
*Tet1* deficiency inhibits the expression of GluA1 and reduces Ca^2+^ signaling. **(A)** 5hmC-seq data analysis identified three loci on GluA1 showing significant 5hmC enrichment for wildtype astrocytes but not for *Tet1* KO astrocytes. **(B)** qPCR results validated the decreased 5hmC enrichment on GluA1. Fold enrichment was calculated as 2-*dCt*, where *dCt* = *Ct* (5-hmC enriched)—*Ct* (input). Data were presented as mean ± SEM, *n* = 3, unpaired *t*-test; **P < 0.01; ***P < 0.001. **(C)** Representative immunostaining images of GluA1 and Gfap with WT and *Tet1* KO astrocytes. Scale bar, 20 μm. **(D–F)** qRT-PCR **(D)** and western blot assay results **(E,F)** showed that *Tet1* KO significantly reduced the level of GluA1. Data were presented as mean ± SEM, *n* = 3, unpaired *t*-test; **P < 0.01; ***P < 0.001. **(G,H)** Representative images show the different Ca^2+^ signal intensity between WT and *Tet1* KO astrocyte evoked by 100 nM ATP **(A)**, 1,000 nM **(B)** ATP, respectively, which indicated by Fluo. 8 am. 8–10 cells were analyzed for each group. **(I)** Ectopic GluA1 restored Ca^2+^ signaling of *Tet1* KO astrocytes. WT astrocyte was infected with lentivirus expressing RFP. KO astrocytes were infected lentivirus expressing RFP, and RFP-GluA1, respectively. 8–10 cells were analyzed for each group.

Given *Tet1* deficiency in astrocyte affecting neuronal development and memory, we speculate that *Tet1* deficiency induced the deficits of communication between astrocytes and neurons. RNA-seq data analysis showed that the expression of *GluA1*, the subunit of AMPA receptor, was significantly decreased in *Tet1* KO astrocytes (Supplementary Figure 4A), whereas other subunits of AMPA receptor *GluA2-3* were increased (Supplementary Figures 4B–D). 5hmC-seq results and 5hmC-IP followed by qPCR both showed a significant decrease of 5hmC modification on GluA1 (Figures 5A,B). Immunofluorescence staining of GluA1 with WT and *Tet1* KO astrocytes showed that the signal intensity of GluA1 was significantly decreased in *Tet1* KO astrocytes compared to WT astrocytes (Figure 5C). Consistently, qRT-PCR and western blot assay results showed that the expression of GluA1 was significantly decreased in *Tet1* KO astrocytes (Figures 5D–F). Immunofluorescence staining results showed that the signal intensity of GluA1 was significantly decreased in hippocampi region of *Tet1* cKO mice compared to WT mice (Supplementary Figure 4E). In addition, qRT-PCR and western blot assays showed that acute knock down of *Tet1* also decreased the expression of *GluA1* (Supplementary Figures 4F–H). Taken together, these results suggest that *Tet1* deletion reduced the expression of GluA1 in astrocyte. Considering the important function of GluA1 in Ca^2+^ signaling, we next tested Ca^2+^ signal in WT and KO astrocytes with ATP administration. We found that *Tet1* KO astrocyte almost completely lost response to ATP treatment at concentrations of 100 and 1,000 nM (Figures 5G,H).

Finally, we examined whether ectopic GluA1 could rescue the deficits of Ca^2+^ signal in *Tet1* KO astrocytes. Western blot assay results showed a high expression efficiency of lentivirus vector expressing GluA1 in N2a cells (Supplementary Figures 4I,J). We then infected the cultured WT and *Tet1* KO astrocyte with lentivirus expressing RFP, and RFP + GluA1, respectively, followed by the treatment with 1,000 nM ATP. We observed that ectopic GluA1 could significantly restored Ca^2+^ signal (Figure 5I and Supplementary Figure 4K). Taken together, these results suggest that *Tet1*-loss induced the deficits Ca^2+^ signaling can be rescued by ectopic GluA1 in astrocyte.

## Discussion

Although previous studies have revealed the function of Tets in neurons, in neural stem cells and in cognitive function, the role of Tet in astrocytes still remains unknown (Kaas et al., 2013; Rudenko et al., 2013; Zhang et al., 2013; Zhu X. et al., 2016; Li X. et al., 2017). In the present study we focused on the physiological function of DNA dioxygenase Tet1 in mouse astrocytes. We found that astrocytes *Tet1* loss significantly decreased the global level of 5-hydroxymethylcytosine (5hmC). Specific ablation of Tet1 in astrocytes impaired learning and memory of adult mice and neuronal development. 5hmC genome sequencing showed that *Tet1* deletion induced differentially hydroxymethylated regions (DhMRs), and RNA-seq results showed that *Tet1* loss altered gene expression. Finally, we revealed that *Tet1* deficiency in astrocytes resulted in the abnormal Ca^2+^ signaling of astrocytes by modulating the expression of GluA1. Taken together, these data suggest that Tet1 is important for the function of astrocytes.

Previous research revealed that modulation of Tet1 and Tet3 affected neuronal activity, gene expression, and consequently regulated memory formation and extinction and the formation cerebellar circuitry (Guo et al., 2011; Kaas et al., 2013; Rudenko et al., 2013; Yu et al., 2015; Zhu X. et al., 2016). Although these studies identified the critical function of Tet1 andTet3 in the neuronal system, the evidence regarding the role of Tet in astrocytes is still lacking. Our results showed that specific ablation of astrocytes Tet1 not only significantly decreased the level of 5hmC and altered the morphology of astrocytes, it also impaired neuronal development and cognitive function of mice. Therefore, on the one hand, our findings, suggest that Tet1 plays important functions in different types of cells including neurons and astrocytes. On the other hand, our study provides a new layer of mechanism for how Tet1 regulates brain function, especially for explaining the *in vivo* data generated with *Tet1* constitutive knockout mice (Rudenko et al., 2013; Zhang et al., 2013).

Tets, including Tet1-, Tet2- and Tet3-mediated 5hmC, are dynamic and also conservative during neuronal development and neurogenesis (Szulwach et al., 2011; Hahn et al., 2013; Li X. et al., 2017). Both tissue/cell and developmental stage affect 5hmC distribution at distinct genomic regions, and they also affect the acquisition and loss of 5hmC (Szulwach et al., 2011). Our results showed that 5hmC is almost equally enriched at promoter and gene bodies regions in astrocytes and is different from those of neurons and neural stem cells, which are highly enriched at gene bodies (Song et al., 2011; Szulwach et al., 2011). Consistently, 5hmC is further enriched at promoter and gene bodies of up-regulated genes, while being less enriched at promoter and TES regions of down-regulated genes, underlining the concept that 5hmC is positively associated with gene expression (Mellen et al., 2012; Hahn et al., 2013; Li X. et al., 2017).

Astrocyte tightly interacts with neuronal cells and are involved in brain development and disorders (Clarke and Barres, 2013; Sloan and Barres, 2014; Phatnani and Maniatis, 2015; Zuchero and Barres, 2015; Allen and Lyons, 2018; Khakh and Deneen, 2019). Astrocyte expresses ionotropic and/or metabotropic receptors, and the binding of glutamate to ionotropic and/or metabotropic receptors activates glutamate signaling. The activation of glutamate receptors induces the generation of intracellular ion signals and/or second messengers including ATP release and calcium signaling in astrocytes (Rose et al., 2017). Astrocyte calcium signaling is not only an essential feature of astrocyte activity, also an important mechanism for neuron-glia interaction at synapses. Aberrant calcium signaling is involved in neurodevelopmental and neurodegenerative diseases (Robel and Sontheimer, 2015; Verkhratsky, 2019). Epigenetic modifications have been shown to regulate gene expression and functioning of astrocytes in both development and diseases (Neal and Richardson, 2018; Puri, 2020). Our study provides evidence that shows that *Tet1* deficiency leads to a significant decrease of GluA1 and impairs astroglial calcium signaling. Our results reveal a new mechanism for how Tet1-mediated 5hmC regulates brain function through affecting astrocyte physiology.

## Materials and Methods

### Animals

Mice were housed in a standard condition of the animal center of Zhejiang University on a 12 h light/dark cycle with free access to food and water. The inducible Tet1 conditional knock out mice (*Tet1*^loxp/lo*xp*^:*Glast-Cre*^ERT2^; cKO) by crossing *Tet1*^loxp/loxp^ mice with Glast-Cre^ERT2^ mice (Jackson Laboratory, #012586). *Tet1*^loxp/loxp^ and *Tet1* constitutive knockout (KO) mice were generated as described previously (Zhang et al., 2013). To induce recombination, adult (7–8 weeks old) mice were injected intraperitoneally with sunflower oil only and with Tamoxifen, respectively (100 mg/kg, 1 time/day for five consecutive days). Tamoxifen was prepared in 10% ethanol mixed with sunflower oil (Wako, #196-15265) with occasional vortexing until completely dissolved. All animal experiments were performed according to the protocols approved by the Institutional Animal Care and Use Committee of Zhejiang University.

### Behavioral Tests

A Morris water maze test was performed as described previously (Li L. et al., 2017). The test was performed in a round, water-filled tub with 120 cm in diameter. After the mice were trained for 6 days, a probe test was performed. All trials were videotaped and were analyzed with MazeScan software (Actimetrica, China). The single time-point data were analyzed by Student’s *t*-test, and the serial days’ data as dependent values were analyzed by two-way ANOVA.

An eight-arm radial maze test was conducted as described previously (Li L. et al., 2017). The apparatus consisted of an octagonal platform at the center and eight identical extending arms equipped with a head-end detector at the end. The movement of the mice was recorded with a video tracking system (Med Associates Inc). The times of the mouse walking through each arm was counted, and data were analyzed by Student’s *t*-test.

### Isolation and Culture of Astrocytes

Neonatal mice (postnatal day 1–3) were sacrificed, and cortical and hippocampi regions were dissected out with a microscope. The tissues were digested with 0.25% trypsin (Gibco, 25200072) for 25 min at 37°C to dissociate into single cell suspensions. About 1 × 10^7^ cells were plated onto one poly D-lysine coated T25 culture flank with DMEM medium supplemented with 10% FBS, 1% antibiotic-antimycotic, 2 mM L-glutamine, and the medium was replaced every 2 days. After cultured 7–10 days, samples were put on a shaker (240 rpm) for 12 h at 37°C, and the medium was completely replaced with fresh culture medium.

### Immunofluorescence Staining

Brain sections were washed with PBS for three times and were blocked with PBS-containing 3% goat serum (Vector Laboratories, #) and 0.1% Triton X-100 for 1 h at room temperature. Sections were incubated with primary antibodies overnight at 4°C and were washed with PBS. The following primary antibodies were used: GFAP (), mouse anti-Neuronal Nuclei (NeuN, Millipore, MAB377), DCX (), and GluA1 (Abcam, ab1232). On the second day, the samples were taken out and washed with PBS for 3 times, 5 min/time, followed by incubation with the secondary antibodies for 1 h at room temperature. Fluorophore-conjugated secondary antibody was used: goat anti-mouse Alexa Fluor 568 (Invitrogen, A11031), goat anti-rat Alexa Fluor 568 (Invitrogen, A11077), goat anti-rabbit Alexa Fluor 488 (Invitrogen, A11008), and goat anti-mouse Alexa Fluor 488 (Invitrogen, A11001). All the sections were observed and images were taken with a confocal microscope (Leica). The images were analyzed with Imarus software.

### Golgi Staining

Golgi staining was performed with FD Rapid GolgiStain Kit according to the manufacturer’s protocol (FD NeuroTechnologies, #). The morphology of CA1 Neurons of adult control and Tet1 cKO mice were analyzed. The dendritic length, numbers of spines, and sholl analysis were analyzed using Image J software.

### Genomic DNA Preparation and Dot Blot

The preparation of Genomic DNA was performed as described previously (Szulwach et al., 2011; Li X. et al., 2017). Briefly, astrocytes were collected, and pellets were lysed with 600 μl DNA lysis buffer (100 mM Tris–HCl, pH 8.0, 5 mM EDTA, 0.2% SDS and 200 mM NaCl) containing Proteinase K and RNase A overnight at 55°C. The second day, equal volumes of phenol:chloroform:isoamyl Alcohol (25:24:1, Sigma, P-3803) were added and completely mixed, followed by centrifugation at 12,000 *g* for 30 min. The supernatant was collected and mixed with 500 μl isopropanol to precipitate DNA. DNA pellets were washed with 70% ethanol and were dissolved with DNase free water. 5-hmC dot blot was performed as described previously (Szulwach et al., 2011; Li X. et al., 2017).

### Western Blot

Cell pellets were lysed with RIPA buffer for 30 min on ice. After centrifugation at 12,000 *g* for 30 min at 4°C, the supernatants were collected. The protein concentrations were measured with a biophotometer (Eppendorf). 20 μg total proteins of each sample were applied for SDS-PAGE electrophoresis; then the gel was transferred to PVDF membranes. The following primary antibodies were used: anti-GAPDH (Ambion, AM4300), anti-GluA1 (Abcam, ab1232), anti-Flag (Thermo, MA1-91878), and anti-HA (Diagbio, db5297). The images were measured by Molecular Imager Imaging System (Tanon, China). The intensity of images was analyzed with Adobe Photoshop software.

### Total RNA Isolation, Quantitative Real-Time PCR, and RNA-Seq

Total RNA was extracted from cultured astrocytes after using TRIzol reagent following the manufacture’s protocol and was purified with chloroform. The concentration of RNA was quantified using a NanoDrop spectrophotometer 2000 (Thermo Fisher Scientific). 0.4 μg of total RNA was used for reverse transcription using a RT reagent kit (Vazyme). Standard real–time qPCR assays were performed using SYBR Green (Vazyme) in triplicate, and the results were analyzed using the ^ΔΔ^Ct method.

All samples used for the cDNA library was assessed with a NanoDrop spectrophotometer 2000, and the RNA integrity value (RIN) was determined with an Agilent 2100 Bioanalyzer (Agilent Technologies Inc.). The extracted mRNA was fragmented, reverse transcribed into cDNA, and ligated with proprietary adapters to the 3′ and 5′ termini. Subsequently, paired-end sequencing was performed with the Illumina HiSeq sequencing technology (Illumina). Raw sequencing output was filtered, and the retained clean reads were then aligned to the *Mus musculus* reference genome (mm10).

### Gene Ontology Analysis

Gene ontology (GO) analysis was performed using the DAVID database (Dennis et al., 2003) as described previously (Chen et al., 2019).

### Co-immunoprecipitation

The cultured astrocytes were collected, washed with PBS, and lysed with RIPA buffer on ice for 30 min, followed by treatment of extraction buffer (Thermo Fisher) containing protease inhibitor cocktail (Roche). The samples were sonicated and centrifuged at 12,000 *g* for 10 min at 4°C. The supernatants were collected and treated with DNase (30 units/ml, Promega) and with RNase A (25 mg/ml) for 20 min at 37°C. The supernatants were incubated with primary antibodies overnight. On the second day, protein A magnetic beads (Sigma) were mixed with samples for 2 h at 4°C. After washing with washing buffer for three times, the beads were re-suspended using 30 μl RIPA buffer and a 10 μl 4X loading buffer. After denaturation, the samples were analyzed using immunoblotting assays, and the second antibodies were used to detect the target proteins.

### 5-hmC Genome-Wide Sequencing and qPCR

The enrichment of 5-hmC of Genomic DNA was performed as described previously (Szulwach et al., 2011). After purification, biotin-5-N_3_-gmC-containing DNA was used for library construction following the Illumina protocol for “preparing samples for ChIP sequencing of DNA.” The sequencing data of 5-hmC were analyzed and DhMRs were identified. For the validation of 5hmC enrichment, input or 5-hmC-enriched DNA was used in triplicate 20-μl qPCR reactions. The sequences of used primers were: peak #1: chr11:56821357-56821456,FW-GGTTCTGTGTTGCCGTAAGC,RV-TGGACTGATAGAAGCC AGGGA; peak #2: chr11: 56823323-56823404, FW-TCATTC AATCACGGGCTCTCA, RV-AGGGAGCGAAACTTGTGAGG. Peak #3: chr11:56824653-56824749, FW-TGGGCCAGTGGAGT GTAGAA, RV-ATAGCCCTGGATTCACCAGC.

### Calcium Imaging

Fluo8 am (Abcam, ab142773) was used to measure the calcium wave of astrocyte (ref). Briefly, astrocyte was plated on the coverslip at a low confluency. After cultured for 48 h, cells were washed with Hanks’ Buffer with 20 mM HEPES and were incubated with 10 μM Fluo8 am for 45 min at room temperature. The calcium signal was evoked by applying ATP, and images were made by confocal microscope (Leica) and were analyzed with a Leica analysis system.

For the rescue of GluA1, WT and Tet1 KO astrocytes were cultured, purified, and plated onto coverslips at a density of 5 × 10^3^/well in 24-well plates. 24 h later, the cells were infected with lentivirus expressing RFP and GluA1, respectively (30 MOI). 2 h later, the medium was replaced with fresh culture medium, and calcium signal was examined 96 h later.

### Quantification and Statistical Analysis

All data are expressed as mean ± SE. GraphPad Prism (GraphPad Software Inc.) was used for statistical analysis. Unpaired Student’s *t*-test was used to determine the differences between two groups; a two-way ANOVA followed by Tukey’s *post hoc* test was used to determine differences between multiple groups. *P* < 0.05 was considered statistically significant.

## Data Availability Statement

The datasets presented in this study can be found in online repositories. The names of the repository/repositories and accession number(s) can be found below: NCBI GEO; GSE164025, GSE165370.

## Ethics Statement

The animal study was reviewed and approved by the Institutional Animal Care and Use Committee of Zhejiang University.

## Author Contributions

XL conceptualized the project and wrote the manuscript. WX, XZ, FL, and YC did astrocytes isolation and culture. YC did immunofluorescence staining, qRT-PCR, western blot and Ca^2+^ signal measurement with the help of JnZ. WQ performed the RNA-seq data analysis. YB and CS maintained the animals and did tamoxifen injection, performed the quantification of immunofluorescence staining and behavioral tests. ZL, YC, and XL analyzed the 5hmC sequencing data. All authors reviewed and approved the final manuscript.

## Conflict of Interest

The authors declare that the research was conducted in the absence of any commercial or financial relationships that could be construed as a potential conflict of interest.

## Publisher’s Note

All claims expressed in this article are solely those of the authors and do not necessarily represent those of their affiliated organizations, or those of the publisher, the editors and the reviewers. Any product that may be evaluated in this article, or claim that may be made by its manufacturer, is not guaranteed or endorsed by the publisher.

## Supplementary Material

Supplementary Figure 1 **(A)** Representative immunostaining images of different astrocyte markers including Aldh1l1 and Glast, and neuron markers including Map2 and Tuj1. Scale bar, 50 μm. **(B)** Quantification results showed the homogeneity of the cultured astrocytes. **(C)** qRT-PCR results showed a high knockout efficiency of *Tet1* in astrocytes. Data were presented as mean ± SEM, *n* = 3, unpaired *t*-test; **P* < 0.05; ***P* < 0.01; ****P* < 0.001. **(D)** Representative images of methylene blue staining of 5-hmC dot blot. **(E,F)** The average swimming speed **(E)** and swimming distance **(F)** during Morris water maze of Ctrl and *Tet1* cKO mice. Data were presented as mean ± SEM, WT = 9, Tet1 cKO = 11, unpaired *t*-test; **P* < 0.05; ***P* < 0.01; ****P* < 0.001. **(G,H)** Eight-arm maze test results showed that cKO mice displayed higher error ratios for both working memory **(G)** and reference memory **(H)** compared to Ctrl mice. Data were presented as mean ± SEM, WT = 9, *Tet1* cKO = 11, unpaired *t*-test; **P* < 0.05; ***P* < 0.01; ****P* < 0.001.

Supplementary Figure 2 **(A)** Representative immunofluorescence staining images of s100β and NeuN with the brain sections of Ctrl and Tet1 cKO mice. Scale bar, 50 μm. **(B)** Quantification results showed the reduced signal intensity of s100β staining of Tet1 cKO mice compared to that of Ctrl mice. Data were presented as mean ± SEM, *n* = 3, unpaired *t*-test; **P* < 0.05; ***P* < 0.01; ****P* < 0.001. **(C)** Schematic illustration of astrocyte-neuron co-culture system. **(D)** Representative GFAP immunofluorescence staining images of astrocytes before and after co-culture with WT neurons. Scale bar, 30 μm. **(E,F)** The average of area **(E)** and perimeter **(F)** of astrocytes before and after co-culture with neurons. Data were presented as mean ± SEM, *n* = 3, unpaired *t*-test; **P* < 0.05; ***P* < 0.01; ****P* < 0.001.

Supplementary Figure 3 **(A)** FPKM of astrocyte cell markers *Gfap*, *Aldh1l1*, and *s100*β, neural stem/precursor cell marker *Nestin*, neuronal progenitor cell marker *DCX*, and neuronal cell marker *Map2* and *Tuj1*. **(B,C)** The distribution of 5hmC enrichment in the genome of WT and *Tet1* KO astrocytes. 5hmC is highly enriched at introns, intergenic regions, exons and promoter regions in the genome of both WT and *Tet1* KO astrocytes. **(D,E)** The distribution of differentially 5hmC modified regions (DhMRs) in the genome of WT and *Tet1* KO astrocytes. *Tet1* KO-induced DhMRs are highly enriched at introns, intergenic regions, exons and promoter regions in the genome of both WT and *Tet1* KO astrocytes.

Supplementary Figure 4 **(A)** FPKM value of GluA1 of WT and *Tet1* KO astrocytes. **(B–D)** qRT-PCR results showing the expression of *GluA2-4* in WT and *Tet1* KO astrocytes. Data were presented as mean ± SEM, *n* = 3, unpaired *t*-test; **P* < 0.05; ***P* < 0.01; ****P* < 0.001. **(E)** Representative images of GluA1 and Gfap immunofluorescence staining with WT and cKO astrocytes. Scale bar, 100 μm. **(F)** qRT-PCR results showed shRNA against *Tet1* significantly decreased the expression of *Tet1* at mRNA level. Data were presented as mean ± SEM, *n* = 3, unpaired *t*-test; **P* < 0.05; ***P* < 0.01; ****P* < 0.001. **(G,H)** qRT-PCR results **(G)** and western blot assay **(H)** showed that *Tet1* KD reduced the expression of GluA1 at mRNA and protein levels. Data were presented as mean ± SEM, *n* = 3, unpaired *t*-test; **P* < 0.05; ***P* < 0.01; ****P* < 0.001. **(I,J)** Western blot assay **(I)** and quantification results **(J)** validated the expression efficiency of lentivirus-GluA1 plasmid in N2a cells. Data were presented as mean ± SEM, *n* = 3, unpaired *t*-test; **P* < 0.05; ***P* < 0.01; ****P* < 0.001. **(K)** Representative images of Ca^2+^ signaling of lentivirus-RFP infected WT astrocytes, lentivirus-RFP infected *Tet1* KO astrocytes, and lentivirus-RFP-GluA1 infected *Tet1* KO astrocytes indicated by fluo-8. Scale bar, 100 μm.
