# Supplementary figures and images for: Tet1 Regulates Astrocyte Development and Cognition of Mice Through Modulating GluA1
Source: Front Cell Dev Biol. 2021 Oct 28;9:644375. doi: 10.3389/fcell.2021.644375 (PMC8581465; doi:10.3389/fcell.2021.644375)

Supplemental Figure 1

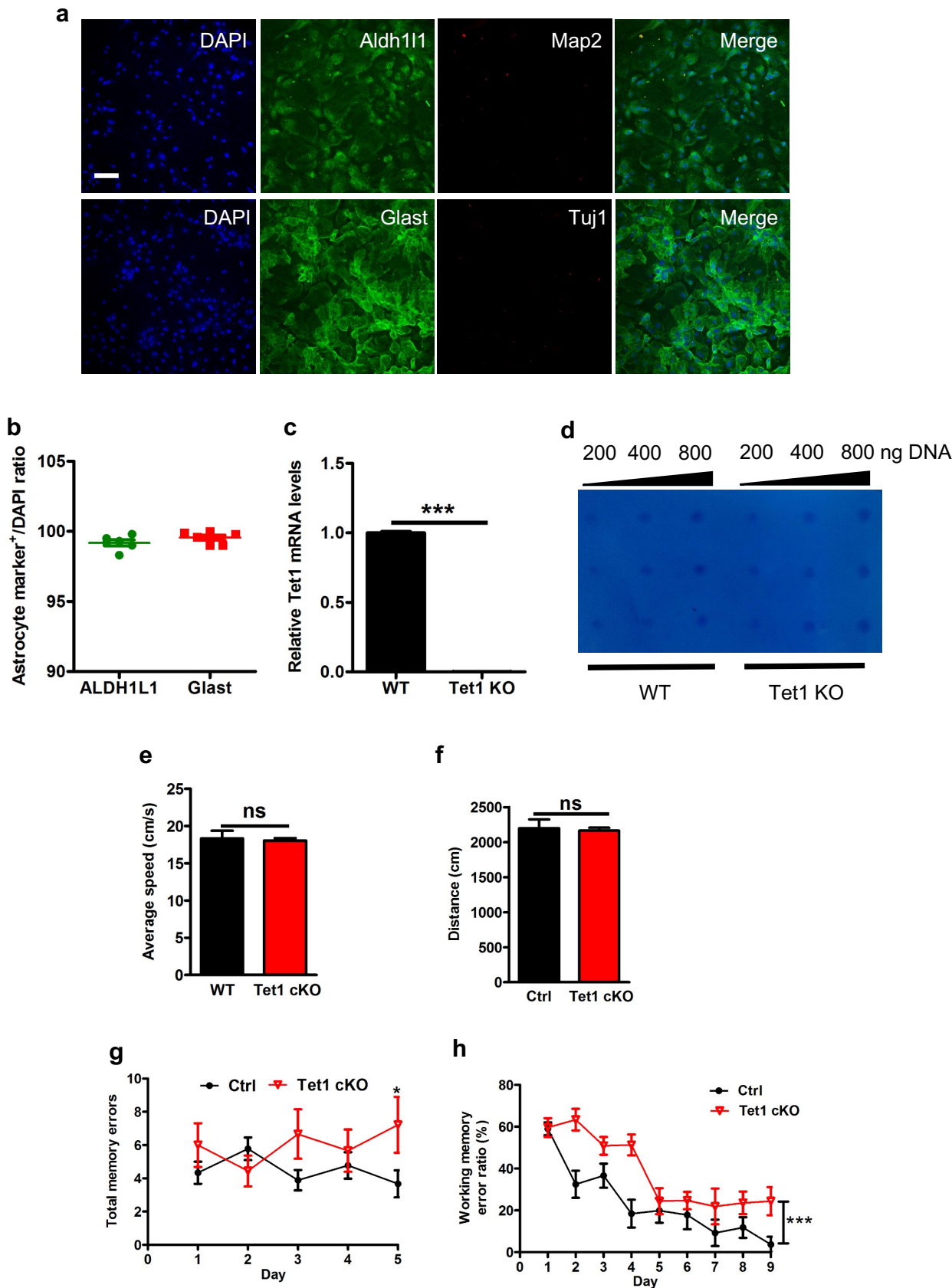

**Supplemental Figure 2**

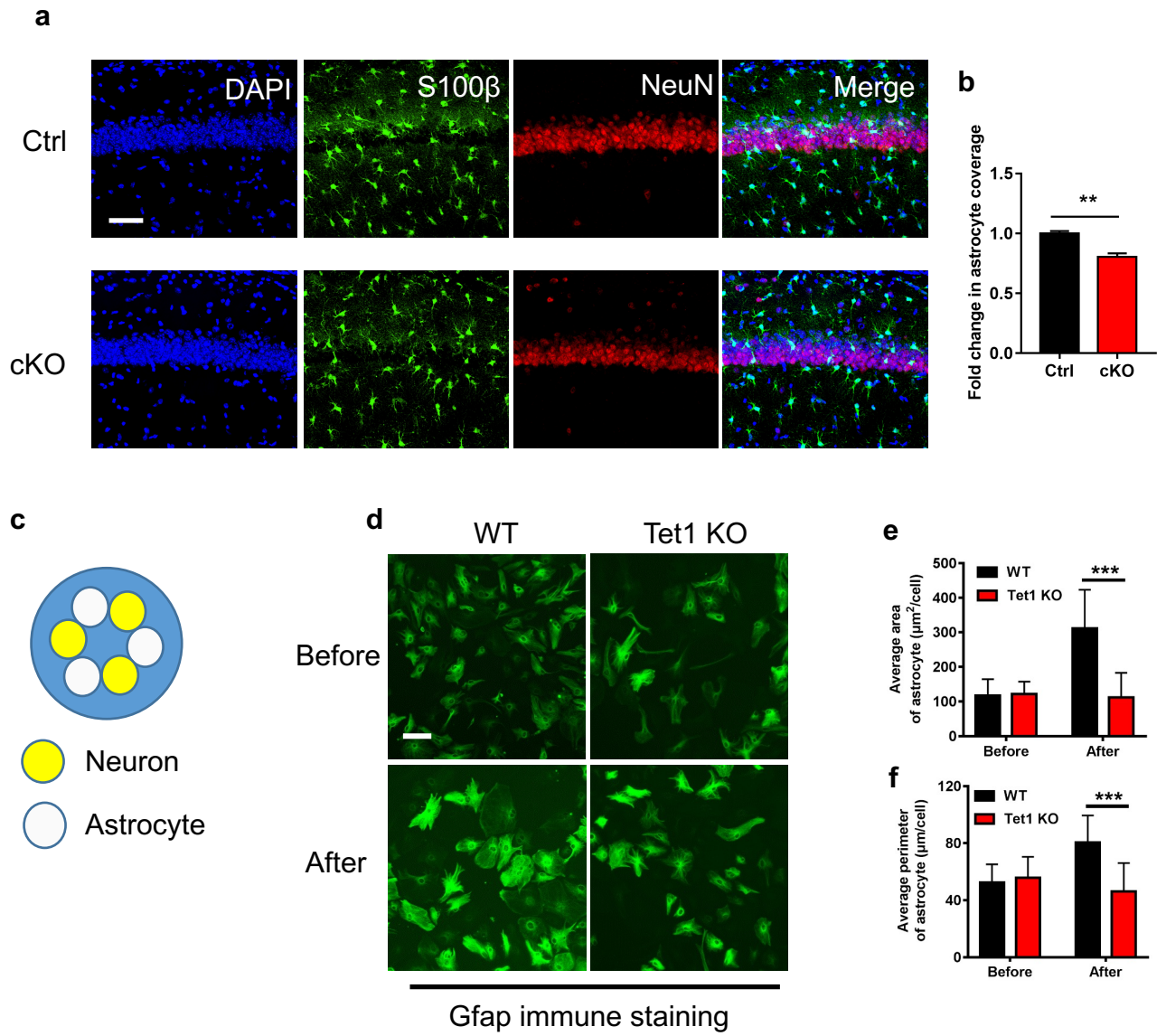

Supplemental Figure 3

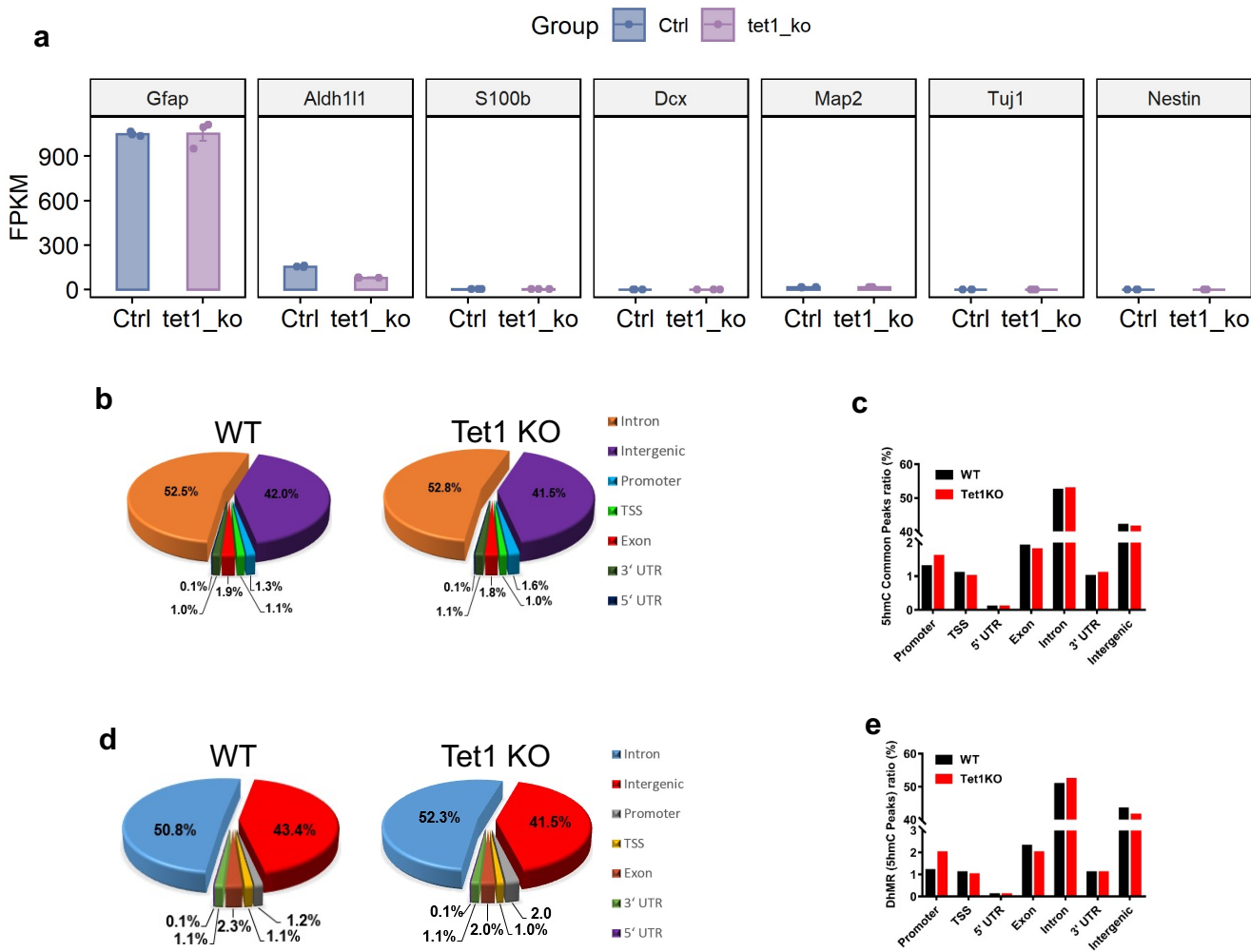

Supplemental Figure 4

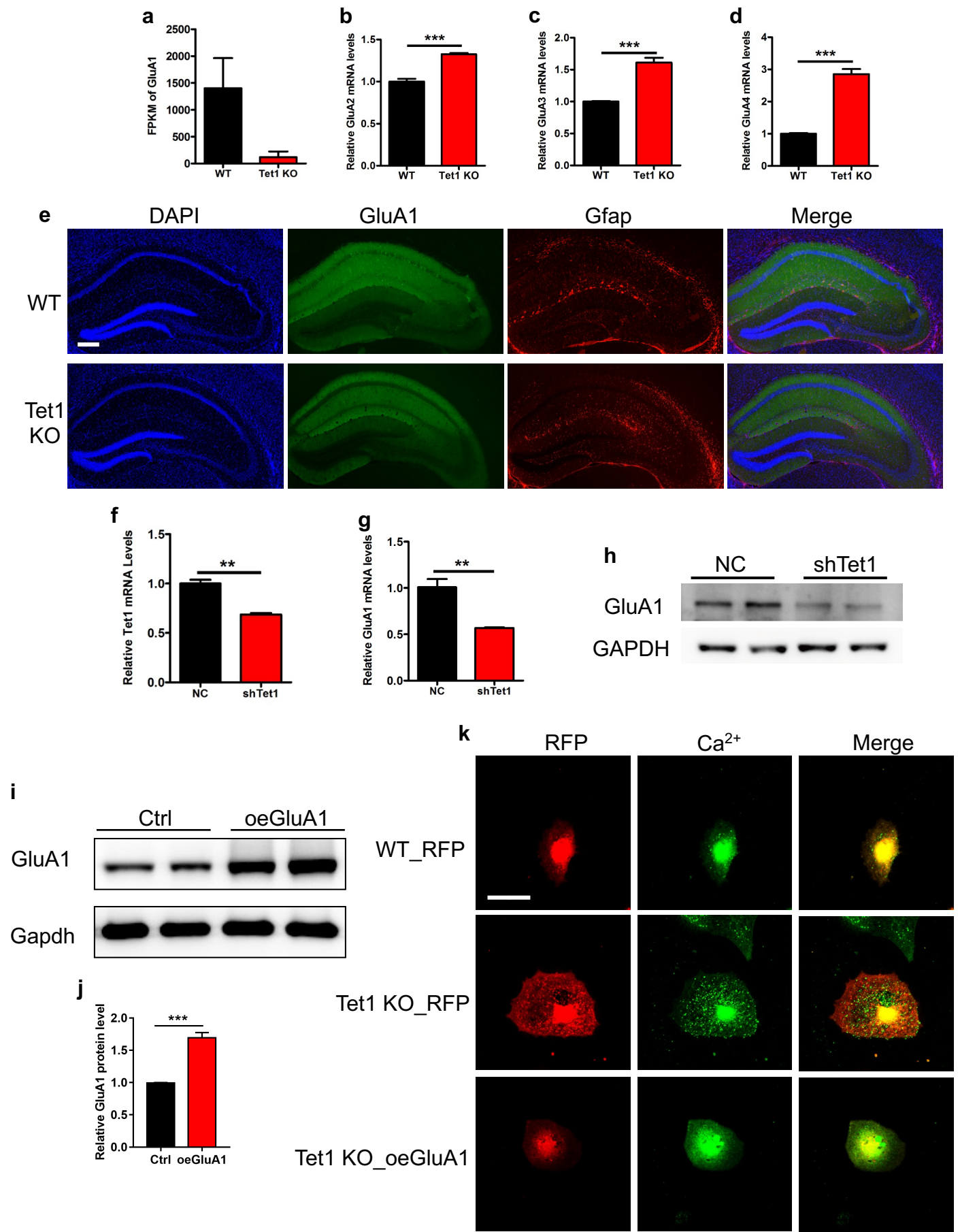

Supplement: Supplementary Figure 1 — (A) Representative immunostaining images of different astrocyte markers including Aldh1l1 and Glast, and neuron markers including Map2 and Tuj1. Scale bar, 50 μm. (B) Quantification results showed the homogeneity of the cultured astrocytes. (C) qRT-PCR results showed a high knockout efficiency of Tet1 in astrocytes. Data were presented as mean ± SEM, n = 3, unpaired t-test; *P < 0.05; **P < 0.01; ***P < 0.001. (D) Representative images of methylene blue staining of 5-hmC dot blot. (E,F) The average swimming speed (E) and swimming distance (F) during Morris water maze of Ctrl and Tet1 cKO mice. Data were presented as mean ± SEM, WT = 9, Tet1 cKO = 11, unpaired t-test; *P < 0.05; **P < 0.01; ***P < 0.001. (G,H) Eight-arm maze test results showed that cKO mice displayed higher error ratios for both working memory (G) and reference memory (H) compared to Ctrl mice. Data were presented as mean ± SEM, WT = 9, Tet1 cKO = 11, unpaired t-test; *P < 0.05; **P < 0.01; ***P < 0.001. [file Data_Sheet_1.PDF]
